# The Role of *Puccinia polysora* Underw Effector PpEX in Suppressing Plant Defenses and Facilitating Pathogenicity

**DOI:** 10.3390/ijms26073159

**Published:** 2025-03-29

**Authors:** Qiang Su, Xiaofan Qi, Kunyu Li, Wenli Zou

**Affiliations:** State Key Laboratory of Plant Environmental Resilience, College of Biological Sciences, China Agricultural University, Beijing 100193, China; cauqxf_1@163.com (X.Q.); caulky@163.com (K.L.); zwl_cau@163.com (W.Z.)

**Keywords:** *Puccinia polysora* Underw, southern corn rust, effector, PpEX, ZmMPK3

## Abstract

*Puccinia polysora* Underw, the pathogen that causes southern corn rust (SCR), delivers effectors to manipulate host immune responses. However, the mechanisms by which these effectors modulate host defenses are not well characterized. In this study, we found that the *P. polysora* effector PpEX is highly upregulated during infection. PpEX suppresses plant immune responses that are initiated by chitin, including the activation of mitogen-activated protein kinases (MAPKs) and the expression of pathogenesis-related (*PR*) genes. Maize plants transiently expressing *PpEX* exhibited higher pathogen infection rates, larger colony areas, and greater fungal biomass on their leaves compared to the control group. By employing TurboID proximity labeling technology coupled with mass spectrometry analysis, we discovered potential target proteins of PpEX in maize. The split-luciferase system enabled us to identify ZmMPK3, a component of the MAPK signaling pathway, as an interacting partner of PpEX among the candidate proteins. This interaction was subsequently confirmed by co-immunoprecipitation (Co-IP) experiments. Additionally, we verified that ZmMPK3 plays a positive role in regulating maize resistance to SCR. Thus, PpEX may function as a virulence effector that dampens plant PTI immunity by interacting with ZmMPK3 and impeding the MAPK signaling pathway.

## 1. Introduction

Rust fungi from the Uredinales order in the phylum Basidiomycota are major plant pathogens affecting many plant families, such as maize and cereals [1]. *Puccinia polysora*, which causes southern corn rust (SCR), is a significant threat to global maize production, especially in humid regions [2]. SCR has historically led to substantial crop losses and economic impacts [3]. While resistant maize varieties have been used to fight SCR, new virulent strains of *P. polysora* can overcome this resistance, maintaining SCR as a threat to maize and food security [3,4]. Thus, understanding the molecular mechanisms of *P. polysora* pathogenicity is crucial for developing durable disease management strategies.

In the ongoing co-evolution with pathogens, plants have evolved a two-tiered immune response [5]. The first line of defense involves pattern-recognition receptors (PRRs) that detect pathogen-associated molecular patterns (PAMPs), initiating PAMP-triggered immunity (PTI). This response is characterized by the activation of mitogen-activated protein kinases (MAPKs), a burst of reactive oxygen species (ROS), and the expression of pathogenesis-related (*PR*) genes [6,7,8,9]. Pathogens can evade PTI by deploying effectors, which plants counter with resistance proteins that detect avirulence effectors, activating effector-triggered immunity (ETI) [5,10]. This response frequently results in a hypersensitive response (HR), causing localized cell death to confine pathogen expansion [5,10].

Pathogen effectors are crucial in their infection, especially those of rust fungi, which are obligate biotrophs. Rust fungi form haustoria for nutrient uptake and also play a role in the production and release of effectors [11]. A variety of effectors, including AvrM14 from *Melampsora lini* [12], RTP1 from *Uromyces fabae* [13], AvrSr50 and AvrSr35 from *Puccinia graminis* f. sp. *tritici* (*Pgt*) [14,15], along with Hasp98, PstGSRE1, PstSIE1, PSTha5a23, and PsSpg1 from *Puccinia striiformis* f. sp. *tritici* (*Pst*) [16,17,18,19,20], have been implicated in pathogen virulence and modulation of host immune responses. For instance, the *Pst* effectors PstGSRE1 and PstSIE1 are highly induced during early infection, and they compromise host immunity by suppressing ROS burst and increased rust pustules [17,19]. The early-expressed effector PsSpg1 targets the wheat susceptibility protein TaPsIPK1, enhancing its kinase activity to phosphorylate the transcription factor TaCBF1d [20]. This phosphorylation suppresses the transcription of disease-related genes and impedes host resistance [20]. Therefore, early-stage effector proteins are essential for rust fungi virulence. Recently, two effectors in *P. polysora* were identified that are highly expressed during infection [4,21,22]. The *AvrRppC* polymorphism in *P. polysora* allows pathogens to circumvent plant recognition via the *RppC* resistance gene, yet the pathogenic mechanism of AvrRppC remains unclear [4]. AvrRppK modulates plant immunity by affecting ROS production and MAPK activation in susceptible maize. However, no specific maize protein target for AvrRppK has yet been found [21]. Liang et al. (2023) examined the transcriptional profiles of *P. polysora* and identified 157 and 338 candidate effector proteins in its A and B haplotypes, respectively, thereby enhancing our understanding of the early effector protein expression profiles of *P. polysora* [22]. However, knowledge of *P. polysora* effectors modulating maize immunity and pathogenicity remains limited.

Effectors frequently target pivotal elements within plant immune signaling pathways [23]. The communication of immune signals through MAPK pathways is pivotal for plants’ natural defense mechanisms, including the production of phytoalexins and the activation of resistance genes [24,25,26]. However, this pathway is a common target for manipulation by pathogenic effectors from bacteria, oomycetes, and fungi [16,18,23,26]. For instance, HopAI1 from *Pseudomonas syringae* and its *Salmonella* counterpart, SpvC, can deactivate MPK6, enhancing the pathogens’ virulence [26]. Similarly, HopF2 interferes with the MAPK signaling cascade by inhibiting MKK5’s kinase function, preventing the activation of MPK3/6 [27]. Hasp98 from *Pst* disrupts immune signaling by binding to TaMAPK4, which disrupts phytohormone signaling, thereby modulating the plant’s immune response [18]. However, to date, no maize target proteins for *P. polysora* effectors have been identified. Therefore, further identification and functional analysis of *P. polysora* candidate effectors and their target proteins are essential for deepening our understanding of *P. polysora*’s pathogenic mechanisms.

In this study, we identified a highly induced candidate effector, PpEX, from transcriptome sequencing and investigated its function in plants. We found that PpEX interacts with ZmMPK3, a maize protein and homolog of *Arabidopsis thaliana* MPK3. Our findings suggest that PpEX is crucial for suppressing plant defenses and enhancing the pathogenicity of *P. polysora*.

## 2. Results

### 2.1. Characteristics of the Candidate Effector PpEX

**Figure 1:**
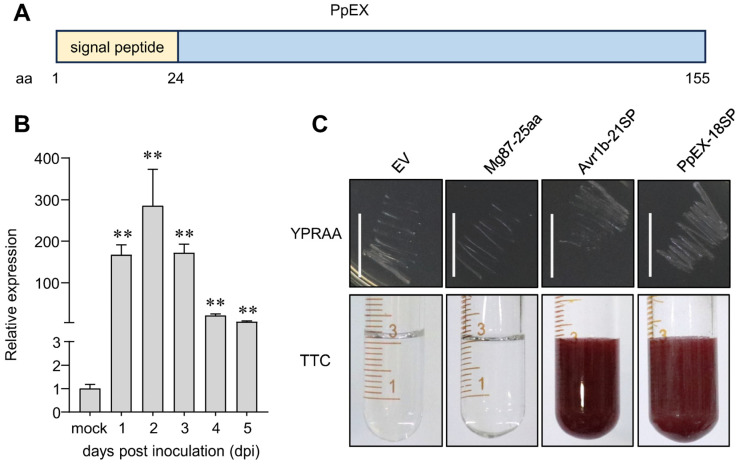
The secreted protein PpEX is highly expressed during *P. polysora* infection. (**A**) Primary structure features of PpEX are shown. (**B**) Quantitative analysis of PpEX transcripts was performed using RNA extracted from germinated spores as a control and from leaves of the B73 maize cultivar infected with GD1913 urediniospores at various time points post-inoculation (1, 2, 3, 4, 5 dpi). *P. polysora* tubulin served as an endogenous control. The expression ratio in germinated spores was normalized to a value of 1. The presented data represents the average with the standard error of the mean from three independent biological replicates. Significance was assessed using an unpaired two-tailed Student’s *t*-test, where ** indicates *p* < 0.01. (**C**) Confirmation of the signal peptide activity of PpEX was carried out by inserting the 21 aa signal peptides from Avr1b, 18 aa putative signal peptides PpEX, and the first 25 amino acids of the non-secretory Mg87 into the pSUC2 vector, which was then introduced into the yeast strain YTK12. Yeast colonies were grown on YPRAA medium with raffinose. Invertase secretion was detected by monitoring the reduction of 2,3,5-triphenyltetrazolium chloride (TTC); a red color change indicates active invertase. Bar = 1 cm.

FUNB_012456-T2, predicted to encode a secreted protein, shows high expression in the *P. polysora* secretome during infection [22]. This protein is only 155 amino acids (aa) long and lacks any known sequence motifs associated with enzymatic function (Figure 1A). To track the transcript expression pattern of FUNB_012456-T2 following *P. polysora* infection, we used qRT-PCR to assess its transcription levels. FUNB_012456-T2 was significantly induced at the early stages of infection, peaking at 2 days post-inoculation (dpi) (Figure 1B). This coincides with the establishment of the parasitic relationship between *P. polysora* and maize. Thus, we designated FUNB_012456-T2 as PpEX.

To confirm if PpEX is a secretory protein, we conducted a yeast signal sequence trap assay to test the secretion capability of its predicted N-terminal signal peptide. The PpEX signal peptide sequence (PpEX-18SP) was inserted into the yeast vector pSUC2 and introduced into the invertase-deficient yeast strain YTK12 [28,29,30,31]. Transformed yeasts were cultured on YPRAA medium with raffinose, which selects yeasts capable of secreting invertase. Invertase activity was determined via the conversion of 2,3,5-triphenyltetrazolium chloride (TTC) to the red compound TPF (1,3,5-triphenylformazan) [31,32]. Yeast strains expressing PpEX-18SP and the positive control Avr1b-21SP showed robust invertase activity, grew on YPRAA medium, and converted TTC to red TPF, unlike the negative control (Mg87-25aa), confirming the functionality of PpEX’s signal peptide in directing protein secretion (Figure 1C). This result verified that the putative N-terminal signal peptide of PpEX is effective in protein secretion.

### 2.2. Subcellular Localization of PpEX in Maize

**Figure 2:**
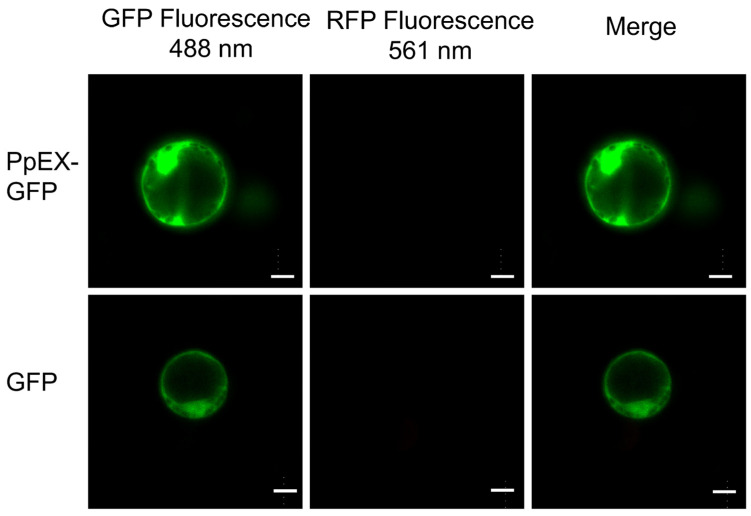
Subcellular localization of the PpEX-GFP fusion protein was performed using protoplasts from maize etiolated seedlings, which exhibited no spontaneous fluorescence under RFP conditions. Bar = 10 μm.

Effectors, once secreted by pathogens, can penetrate host plant cells and may be directed to various intracellular locations [33]. To ascertain the precise subcellular compartment where PpEX resides, the open reading frame (ORF) of PpEX, without the signal peptide sequence, was subcloned and fused with the GFP tag in the expression vector pUC19-35S-GFP and then delivered to maize protoplasts. The transient expression of the PpEX-GFP chimeric protein in maize protoplasts revealed that no spontaneous RFP fluorescence was detected because there were no chloroplasts in the etiolated seedlings’ protoplasts. Consequently, the GFP fluorescence signal was predominantly confined to the cell membrane, nucleus, and cytoplasm (Figure 2).

### 2.3. Overexpression of PpEX in Nicotiana benthamiana Suppresses Programmed Cell Death

**Figure 3:**
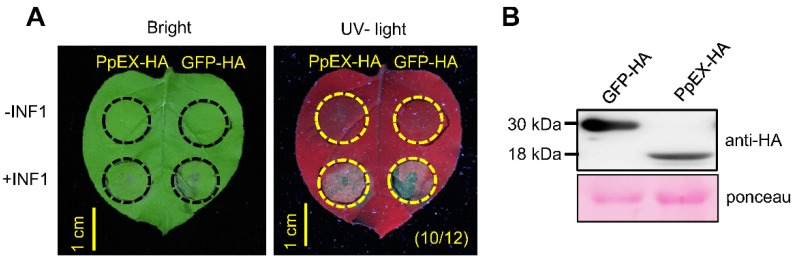
PpEX suppresses INF1-triggered cell death. (**A**) PpEX suppresses INF1-triggered cell death in *N. benthamiana* leaves. *Agrobacterium* suspensions (OD600 of 0.2) carrying GFP-HA and PpEX-HA were introduced into the leaf tissues 24 h prior to INF1 infiltration (left panel). Images were captured 3 days after the INF1 treatment. The corresponding leaves were also visualized under ultraviolet illumination (right panel). The circle represents the injection area. (**B**) Western blot analysis confirmed the expression of PpEX-HA and GFP-HA.

Assessing the ability to inhibit programmed cell death (PCD) induced by the *Phytophthora infestans* PAMP INF1, which is analogous to the plant HR related to defense, serves as a relevant method to gauge the virulence function of effectors [33,34,35,36]. We investigated whether overexpressing *PpEX* in *N. benthamiana* could inhibit PCD triggered by INF1 to evaluate the virulence function of this candidate effector. The results showed that overexpressing *PpEX* inhibited PCD induced by INF1, an effect not observed with the overexpression of GFP as a control (Figure 3A). The presence of PpEX-HA and GFP-HA proteins in the infiltrated tissues was confirmed using Western blot (Figure 3B). These results suggest that the transient expression of *PpEX* suppresses chitin-triggered immunity in *N. benthamiana*.

### 2.4. PpEX Suppresses Maize PTI Immunity

**Figure 4:**
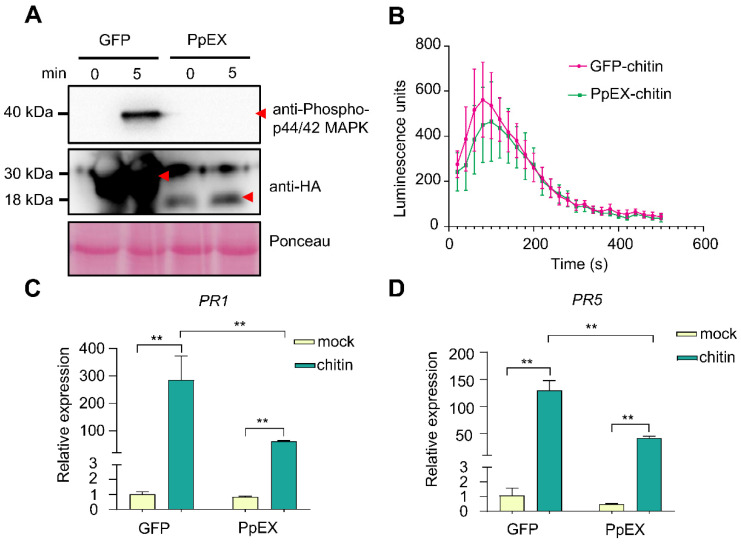
The inhibitory effect of PpEX on the maize immune response. Maize plants infected with SCMV-PpEX were inoculated with chitin, and the inhibitory effect of the effector protein PpEX on MAPK activation (**A**), ROS burst (**B**), *PR1* (**C**), and *PR5* (**D**) gene expression was then detected. The ** (*p* < 0.01) indicates significant differences between treatments and WT, as determined by Student’s *t*-test. Error bars represent the standard deviation. The red arrow represents the target protein.

To evaluate PpEX’s impact on the innate maize immune system, we transiently expressed *PpEX* alongside GFP as a control in maize leaves using *Sugar Cane Mosaic Virus* (SCMV) [37]. The data indicated that plants transiently expressing *PpEX* showed reduced MAPK kinase activation in response to chitin compared to control plants (Figure 4A). Additionally, qRT-PCR analysis revealed that the transcript levels of PTI-associated marker genes *ZmPR1* and *ZmPR5* were downregulated in leaves expressing PpEX-HA relative to those expressing GFP (Figure 4C,D). However, the accumulation of chitin-induced ROS did not significantly differ between PpEX-expressing and control plants (Figure 4B). These findings indicate that the transient expression of *PpEX* in maize suppresses chitin-induced immune responses.

### 2.5. Overexpression of PpEX Enhances P. polysora Pathogenicity in Maize

**Figure 5:**
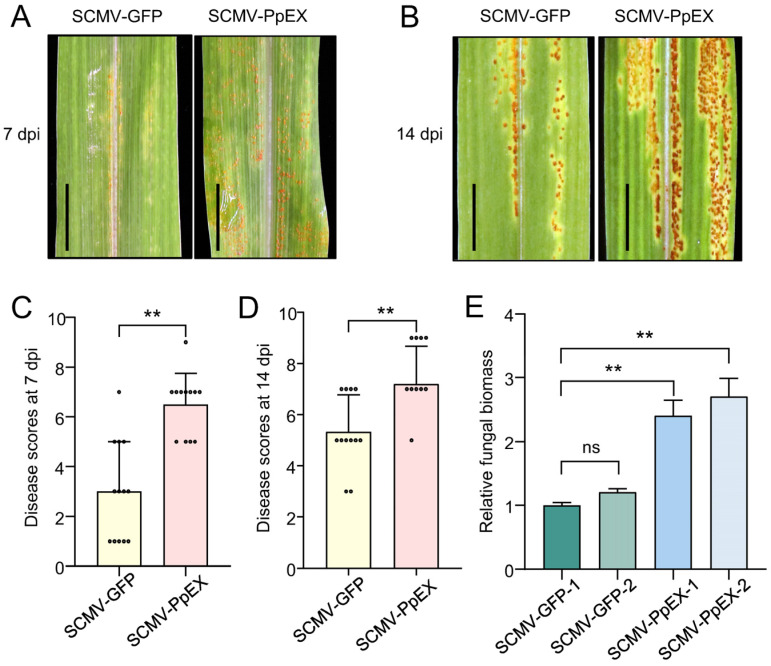
PpEX promotes infection of *P. Polysora* on maize. Maize plants infected by SCMV-GFP and SCMV-PpEX were inoculated with *P. polysora* GD1913. After 7 days of inoculation, the disease grades (**A**,**C**) and fungal biomass (**E**) were assessed. After 14 days of inoculation, the disease grades (**B**,**D**) were assessed. The ** (*p* < 0.01) indicates significant differences between treatments and the wild type (WT), as determined by Student’s *t*-test. The small circles in the bar chart represent the number of maize samples in the experiment. Error bars represent the standard deviation. Bar = 1 cm.

After inoculation with *P. polysora*, plants with transient expression of *PpEX* showed increased susceptibility to SCR at 7 and 14 dpi compared to WT plants expressing *GFP* (Figure 5A–D). A higher accumulation of *P. polysora* biomass was observed in plants with transient *PpEX* expression than in WT plants (Figure 5E). This suggests that PpEX expression can exacerbate SCR development. The findings imply that PpEX suppresses immunity triggered by *P. polysora*, thereby facilitating *P. polysora* infection in maize.

### 2.6. PpEX Interacts with the Maize ZmMPK3

To explore the mechanism underlying PpEX’s role in suppressing plant immunity, we employed a TurboID-based proximity labeling approach in maize plant protoplasts to identify proteins interacting with the biotinylated PpEX protein. We initially lysed the protoplast samples and extracted the proteins. After vigorous cell lysis, the protein lysate was passed through a desalting column to remove excess free biotin, ultimately yielding a pure protein supernatant. This supernatant was then incubated overnight with Streptavidin beads and analyzed by Western blot. The analysis revealed a single clear protein band corresponding to PpEX TurboID HA at 70 kDa, detectable using anti-biotin antibodies in the cell lysate, desalted lysate, and on Streptavidin beads (Figure S1). Additionally, anti-HA antibodies detected bands of the recombinant PpEX-TurboID-HA protein at the same location (Figure S1). These findings suggest that the PpEX-TurboID-HA protein is expressed normally. We also used liquid chromatography-tandem mass spectrometry (LC-MS/MS) to identify potential interacting proteins of PpEX in maize cells. Ultimately, we identified seven candidate proteins that may potentially interact with PpEX, including five that are functionally annotated as being associated with PTI in maize.

**Figure 6:**
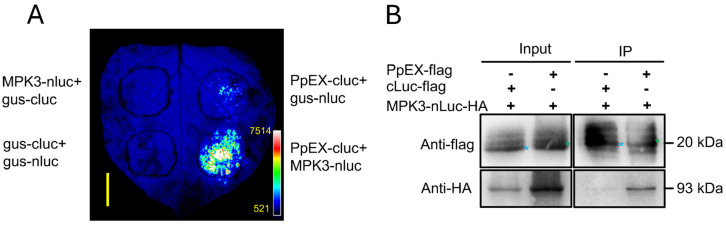
PpEX interacts with ZmMPK3 in vivo. (**A**) Split-luciferase analysis of the interaction between PpEX and ZmMPK3 in *N. benthamiana* leaves. The β-glucuronidase-cLuc (gus-cLuc) and β-glucuronidase-nLuc (gus-nLuc) were used as negative controls. The pseudocolor bar shows the range of luminescence intensity. Bar = 1 cm. (**B**) The interaction of PpEX and ZmMPK3 was detected by co-immunoprecipitation. Total proteins were extracted from leaves co-expressing PpEX-flag and ZmMPK3-nLuc-HA. The input proteins (Input) and proteins extracted with Flag magnetic beads were analyzed by immunoblotting with anti-Flag and anti-HA antibodies. The cLuc-flag protein served as a negative control, with component indicators at the top and protein combinations and antibody indicators on the left. The blue pentagram indicates cLuc-Flag, while the green pentagram indicates PpEX-flag.

To substantiate the interactions, we conducted a split-luciferase analysis to identify potential maize proteins that might interact with PpEX. The expression of fusion proteins was confirmed through immunoblotting. Western blot analysis confirmed the successful transient expression of PpEX, MPK3, MPK6, RAF, CERK1, and RAF1 in *N. benthamiana* leaves, with the exception of RbohD (Figure S2G,H). We found that ZmMPK3-nLuc could interact with PpEX-cLuc and trigger stronger luminescence in the split-luciferase assay, while β-glucuronidase-cLuc (gus-cLuc) and β-glucuronidase-nLuc (gus-nLuc) were used as negative controls (Figure S2B–F). Thus, we selected ZmMPK3 for further study. In an additional split-luciferase assay, luminescence was observed in the interaction of ZmMPK3-nLuc with PpEX-cLuc, and no luminescence was observed in the gus-cLuc/ZmMPK3-nLuc, gus-nLuc/PpEX-cLuc, and gus-cLuc/gus-nLuc groups (Figure 6A). In a co-immunoprecipitation (Co-IP) assay, ZmMPK3-nLuc-HA could be coprecipitated with PpEX-flag, and it did not coprecipitate with the negative control cLuc-flag (Figure 6B). These results validate the interaction between PpEX and ZmMPK3.

### 2.7. ZmMPK3 Positively Regulates Plant Resistance to SCR

Phylogenetic analysis showed that ZmMPK3 is evolutionarily close to ZmMPK6 (NP_001105238.1), *Oryza sativa* OsMPK3 (AAK01710.1), *Arabidopsis* AtMPK3 (NP_190150.1), and *N. benthamiana* NbWIPK (BAC53771.1) (Figure S3), all of which are known to be involved in disease resistance [25,38,39].

**Figure 7:**
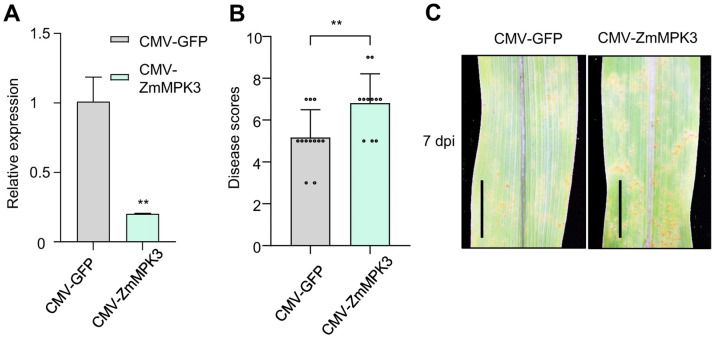
Knocking down *ZmMPK3* with CMV-VIGS decreased resistance to *P. polysora* in B73 maize plants. (**A**) qRT-PCR analysis of *ZmMPK3* expression in CMV-infected B73 plants at 15 dpi. (**B**) Disease scores of CMV-infected B73 plants at 7 dpi with *P. polysora*. The small circles in the bar chart represent the number of maize samples in the experiment. (**C**) Urediospore symptoms on leaves of CMV-infected B73 plants at 7 dpi with *P. polysora*. dpi indicates days post-inoculation. Significance was determined by an unpaired two-tailed Student’s *t*-test (** *p* < 0.01). Error bars represent the standard deviation. Bar = 1 cm.

To determine the role of ZmMPK3, we employed *Cucumber Mosaic Virus*-based Virus Induced Gene Silencing (CMV-VIGS) to silence the *ZmMPK3* gene in maize B73 and subsequently evaluated its disease resistance. The results showed that the expression level of *ZmMPK3* in the silenced plants was reduced to 80% of the wild-type level 15 days after inoculation with CMV-ZmMPK3 (Figure 7A). When challenged with *P. polysora* race GD1913, the *ZmMPK3*-silenced plants exhibited a higher number of urediniospore pustules than the wild-type plants (Figure 7B,C).

## 3. Discussion

Effectors play a pivotal role in plant–pathogen interactions, with numerous studies implicating them as virulent factors that can subdue plant defenses and promote disease development [5,40]. Despite their importance, the role of effectors in *P. polysora* remains largely unexplored. Our research delved into the function of the *P. polysora* candidate effector PpEX, offering valuable insights into the pathogenic mechanisms of *P. polysora*.

Most pathogen virulence effectors suppress the HR associated with PTI during early infection [16,18,23]. For example, *Pst* effectors Hasp98 and PSTha5a23, the *Magnaporthe oryzae* effector MoPtep1, and the *Bursaphelenchus xylophilus* effector BxSCD3 all inhibit PTI-related HR, contributing to virulence [16,18,41,42]. Similarly, PpEX from *P. polysora* suppresses cell death induced by INF1 in *N. benthamiana* (Figure 3A), indicating its role in inhibiting host basal immunity. Plant PRRs detect pathogenic PAMPs, such as fungal chitin and bacterial flg22, trigger plants to produce ROS, activate MAPKs, and express *PR* genes, thereby promoting the occurrence of plant disease resistance responses [9]. Indeed, PpEX diminishes PTI responses, such as MAPK activation and *PR* gene expression (Figure 4A,C,D). The effector AvrRppK compromises maize resistance during incompatible interactions with *P. polysora* [21]. Moreover, overexpression of PpEX in maize reduces resistance to *P. polysora* (Figure 5), suggesting that PpEX is likely to act early in the PTI pathway to avoid plant recognition. Effectors can also inhibit ETI immune receptors and interfere with plant hormone signaling transduction. *P. syringae* AvrPtoB targets plant NLR proteins ADR1-L1 and ADR1-L2, promoting their degradation to suppress ETI [43]. *Ustilago maydis* Cmu1, a chorismate mutase, inhibits SA biosynthesis to reduce plant resistance [44]. Despite lacking a known structural domain, PpEX’s potential to inhibit plant ETI and other immune responses warrants further study. Reports indicate that diverse rust fungi harbor homologous effector proteins with virulent capabilities. PsSpg1 homologs, PgSpg1 and PtSpg1, are found in *Pgt* and *Puccinia Triticinia*, respectively [20]. PtSpg1 and PgSpg1 share the same target protein, which can interact with TaPsIPK1 to inhibit wheat disease resistance [20]. Further investigation of PpEX homologs in other rust fungi is essential for uncovering their role in pathogenicity enhancement and identifying shared targets, thereby enhancing our understanding of rust fungi’s pathogenic mechanisms. Pathogens secrete a plethora of effector proteins at the onset of infection to target and subvert plant immune mechanisms, thereby hijacking the host’s defense response [23]. Given the central role of MAPK pathways in plant innate immunity, these kinases are frequently targeted by effectors from pathogens [26]. In this study, we demonstrated that the *P. polysora* effector PpEX directly interacts with ZmMPK3 (Figure 6). Phylogenetic analysis showed that ZmMPK3 is part of a clade that includes ZmMPK6 (Figure S3), OsMPK3, AtMPK3, and NbWIPK, and numerous studies have established that these kinases play crucial roles in plant resistance to pathogens, underpinning basal resistance [25,38,39]. Silencing *ZmMPK3* decreased maize resistance to SCR, indicating its positive role in defense against SCR (Figure 7). Previous studies have shown that the *Pst* effector Hasp98 directly targets and inhibits the kinase activity of TaMAPK4, which is a positive regulator of wheat’s defense against stripe rust [18]. Given the interaction between PpEX and ZmMPK3, we infer that PpEX might impede plant immune responses by inhibiting the kinase activity of ZmMPK3. Some effectors can target multiple host immune elements to exert their effects. For instance, the *M. oryzae* effector AvrPi-t manipulates plant immunity and facilitates pathogen infection by interacting with five key host proteins: the E3 ubiquitin ligases APIP6 and APIP10 [45,46], the bZIP transcription factor APIP5 [47], the nuclear porin-like protein APIP4 [48], and the potassium channel protein OsAKT1 [49]. Applying these target proteins significantly enhances rice resistance to *M. oryzae* [45,46,47]. Therefore, future research into additional maize target proteins of PpEX may reveal valuable and actionable genetic resources.

Examining effectors’ target proteins is vital for revealing key components of plant immune signaling and providing genetic resources crucial to the development of disease-resistant crop varieties. Indeed, through the use of the *P. syringae* effector protein AvrPphB, the immune components BIK1 and PBL1 were identified in *Arabidopsis*, substantially broadening our understanding of the PTI immune response [50]. In this study, we identified PpEX as a virulence effector that targets ZmMPK3, a kinase implicated in maize resistance to SCR. By examining the target proteins of effectors, researchers have identified a range of new genes in wheat, rice, and soybean that could substantially improve plant resistance to diseases [20,51,52]. For example, overexpression of *TaMAPK4* significantly bolsters wheat resistance to *Pst* [18]. Similarly, overexpression of *ZmWAK17*, a target of the *Fusarium graminearum* effector CFEM1, significantly enhances maize resistance to stem rot [53]. Herein, we demonstrated that *ZmMPK3* is involved in maize resistance to SCR. In future studies, we plan to generate stable transgenic maize expressing *ZmMPK3* and evaluate its disease resistance in the field. This work could provide valuable genetic resources for developing maize varieties resistant to SCR.

## 4. Materials and Methods

### 4.1. Plant Growth and Virus Inoculation

The B73 inbred line of maize and *N. benthamiana* were grown under controlled environmental conditions set at 22 °C with a 16-h light/8-h dark cycle.

The SCMV-GFP and SCMV-PpEX-HA viruses were propagated in *N. benthamiana* seedlings. We collected the inoculated leaves from these seedlings for subsequent inoculations on maize. Healthy maize plants were inoculated with crude extracts from the infected leaves using mechanical transmission, following established methods [37].

The VIGS approach in maize using cucumber mosaic virus was implemented as per previous studies [54]. In essence, a 220-bp segment of the *ZmMPK3* gene was inserted into the pCMVZ22bN81 vector to create the construct pCMVZ22bN81::ZmMPK3, which was designed to silence the *ZmMPK3* gene in maize. This construct was transformed into *Agrobacterium* and infiltrated into *N. benthamiana* leaves. Seven days post-infiltration, the upper leaves were collected and used to inoculate maize kernels, in accordance with standard procedures [54]. Seeds inoculated with CMV were planted in pots, and two weeks post-planting, expression profiling of *ZmMPK3* was conducted on the seedling leaves.

### 4.2. Agrobacterium-Mediated Transient Expression in N. benthamiana

The recombinant plasmids were transferred into *A. tumefaciens* strain GV3101. For the leaf infiltration process, the recombinant *Agrobacterium* strains were grown in a liquid LB medium for a duration of 48 h. They were then collected and re-suspended in an infiltration buffer composed of 10 mM MgCl_2_, 200 μM acetosyringone (AS), and 10 mM MES-KOH (pH = 5.7). This suspension was allowed to incubate at ambient temperature for 3 h before the infiltration procedure. The *Agrobacterium* suspensions, with their optical density adjusted to 0.2 at 600 nm, were applied to the leaves of *N. benthamiana* plants, which were 4 to 6 weeks old, using a syringe devoid of a needle (DONGBEI, Heze, Shandong, China).

### 4.3. Gene Expression Analysis

After treatment, leaf samples were taken at intervals and flash-frozen in liquid nitrogen, with three replicates prepared. Total RNA extraction from the tissues was conducted using a Quick RNA Isolation Kit (Vazyme, Nanjing, Jiangsu, China), and the RNA was reverse transcribed to cDNA with the HiScript^®^ III RT SuperMix for qPCR kits (Vazyme, Nanjing, Jiangsu, China). qRT-PCR was carried out on Thermo Scientific™ Applied Biosystems (ABI) 7500 (Applied Biosystems, Waltham, MA, USA) with ChamQ SYBR qPCR Master Mix (Low ROX Premixed) (Vazyme, Nanjing, Jiangsu, China). Primer sequences are listed in Supplementary Table S1. Gene expression was quantified using the 2^−ΔΔCT^ method [55].

### 4.4. Subcellular Localization

The ORF of PpEX, devoid of its signal peptide sequence, was cloned with primer pairs pUC19-PpEX-GFP-F/R and integrated with the GFP tag within the pUC19-35S-GFP expression vector. The expression of the PpEX-GFP transcript was controlled using the *Cauliflower mosaic virus* (*CaMV*) *35S* promoter. At the two-leaf stage of maize etiolated seedlings, the second leaves were harvested for the isolation of protoplasts. Protoplast extraction and transfection with 10 μg plasmid pUC19-35S-PpEX-GFP via the polyethylene glycol (PEG) 4000–calcium method were performed as described previously [56], followed by protoplast cultivation for 12 h at 25 °C. GFP fluorescence was imaged using a Zeiss 710 microscope with a Fluor ×10/0.50 M27 objective lens (Carl Zeiss AG, Oberkochen, Baden-Württemberg, Germany).

### 4.5. Western Blot Analysis

Protein samples from the maize/tobacco protoplasts or leaves and stored at −80 °C for preservation. Total soluble proteins were isolated following a previously reported plant protein extraction method [57]. Briefly, total protein was extracted using IP Buffer (consisting of 50 mM HEPES-KOH (pH = 7.5), 150 mM KCl, 1 mM EDTA, 0.5% Triton X-100, 100 mM N-Ethylmaleimide, and 1 × complete protease inhibitors). Then, 30 mM DTT-containing loading buffer was added to the protein extraction solution, and then denature the proteins at 100 °C for 5 min. Post-denaturation, the proteins were separated via 10% SDS-PAGE and subjected to Western blot analysis with a 1:4000 dilution of monoclonal antibody. MAPK kinase activity was examined by using anti-phospho-p44/42 antibody (Cell Signaling Technology, Danvers, MA, USA, dilution 1:4000). The blots were then visualized using the Tanon 5200 systerm (Tanon, Shanghai, China).

### 4.6. TurboID Proximity Labeling and Liquid Chromatography-Tandem Mass Spectrometry (LC-MS/MS)

Using a modified biotin labeling protocol based on the TurboID system [58], we performed a proximity labeling assay. Briefly, we cloned the ORF of PpEX without the signal peptide sequence and constructed the pUC19-35S-turboID-PpEX vector. At the two-leaf stage of maize etiolated seedlings, at least 40 leaves were harvested for protoplast isolation. Ultimately, we obtained 10 mL of protoplasts in MMG buffer (containing 15 mmol/L MgCl_2_, 0.1% MES, and 0.6 mol/L D-Mannitol, adjusted to pH 5.7) with a concentration of 2 × 10^5^ cells/mL. We then transformed 320 μg of the pUC19-35S-PpEX-GFP plasmid into maize protoplasts. Subsequently, the maize protoplasts were cultured in 4 mL of W5 medium (containing 154 mmol/L NaCl, 125 mmol/L CaCl_2_, 5 mmol/L KCl, 5 mmol/L glucose, and 0.03% MES, adjusted to pH = 5.7) and supplemented with 40 μM biotin for 14 h.

Afterward, the protoplasts were harvested, and the total protein was extracted. The protein lysate was added to a Zeba Spin Desalting Column (ThermoFisher Scientific, Waltham, MA, USA) and then centrifuged at 1000 rpm and 4 °C to remove excess free biotin, yielding a pure protein supernatant. This supernatant was then incubated overnight with Streptavidin beads to capture biotinylated proteins and the resulting complexes were analyzed by Western blot. The biotinylated proteins were prepared for immediate LC-MS/MS analysis at the public instrument platform of the State Key Laboratory of Plant Environmental Resilience. Peptide processing followed previous methods [59], and the resulting peptide data were searched against the maize protein database. Proteins identified in the control (GFP-TurboID) and those with fewer than two unique peptides were excluded. The remaining proteins were cross-referenced to identify potential maize interactors with over 65% sequence identity using local BLASTp (ncbi-blast-2.14.0).

### 4.7. Yeast Secretion Signal Trapping System

To confirm the signal peptide activity of PpEX, we cloned and inserted the 18-amino-acid putative signal peptide of PpEX into the pSUC2 vector. The first 25 amino acids of the non-secreted Mg87 protein from *M. oryzae* served as a negative control. The YTK12 strain, containing the 21-amino-acid signal peptide from Avr1b, served as the positive control. Subsequently, the modified pSUC2 vectors were introduced into the yeast strain YTK12 using the lithium acetate method [60]. Colonies were selected on CMD-W agar with sucrose (0.67% yeast nitrogen base without amino acids, 0.075% tryptophan dropout supplement, 2% sucrose, 0.1% glucose, 2% agar). Active invertase secretion was assessed on YPRAA medium (1% yeast extract, 2% peptone, 2% raffinose, 2 mg/mL antimycin A), with raffinose as the sole carbohydrate source.

### 4.8. Protein Interaction Analysis

Employing the split-luciferase technique reported previously [61], we agro-infiltrated different combinations of constructs into mature *N. benthamiana* leaves. Post-infiltration for 3 days, the leaves received a 1 mM luciferin solution application. Following a 15-min incubation in the dark, we assessed and captured chemiluminescent signals using the Tanon 5200 system (Tanon, Shanghai, China).

For Co-IP analysis, we followed a slightly modified version of the protocol detailed in reference [4]. Recombinant plasmids were transformed into *Agrobacterium* GV3101 and co-infiltrated into *N. benthamiana* leaves. After 48 h, leaf lysates were prepared using IP Buffer, as described in Section 4.5. The supernatant was incubated with Flag magnetic beads (ThermoFisher Scientific, Waltham, MA, USA) at 4 °C for 60 min, followed by centrifugation at 1000 g to pellet the beads. The beads were then washed four times with the IP buffer, and immune complexes were eluted using 3× FLAG peptide TFA (MedChemExpress, Shanghai, China). These complexes were subsequently analyzed by SDS-PAGE and Western blot.

### 4.9. ROS Measurement

Apoplastic ROS were detected using a luminol-based assay, as previously described [62]. Samples of maize seedling leaves inoculated with SCMV-GFP or SCMV-PpEX were taken using a 3 mm diameter punch (KAI, Gifu Prefecture, Tokyo, Japan) and placed into the ELISA plate. Each sample has eight replicates. The leaves were cultivated in water at 25 °C in the dark for 12 h. Afterward, the water was extracted and replaced with Substrate buffer (2 × L-012, 4 mg/mL chitin). Immediately following this, luminescence was recorded using an En-Spire Multimode plate Reader (Perkin Elmer, Waltham, MA, USA). The signal was measured once per 20 s for a duration of 20 min, and the data were analyzed to observe the fluctuations in ROS production throughout this period. The experiment was conducted at least three times, yielding consistent outcomes. Additionally, statistical analysis was performed using a two-tailed Student’s *t*-test, with a significance level set at *p* < 0.05.

## 5. Conclusions

In this research, we investigated the key virulence effector PpEX from *P. polysora*, which was found to directly suppress plant PTI in maize. PpEX was shown to interact directly with ZmMPK3, a kinase that promotes resistance to SCR. These findings advance our comprehension of the molecular mechanisms underlying the pathogenicity of *P. polysora*.

## Data Availability

Data are contained within the article and Supplementary Materials.

## Supplementary Materials

The following supporting information can be downloaded at: https://www.mdpi.com/article/10.3390/ijms26073159/s1.
