# Supplementary material for: The Role of Puccinia polysora Underw Effector PpEX in Suppressing Plant Defenses and Facilitating Pathogenicity
Source: Int J Mol Sci. 2025 Mar 29;26(7):3159. doi: 10.3390/ijms26073159 (PMC11989160; doi:10.3390/ijms26073159)
Supplement: Supplementary file 1 [file ijms-26-03159-s001.zip › ijms-3308831-supplementary.pdf]

Supplementary data
Supplemental Figures and Legends

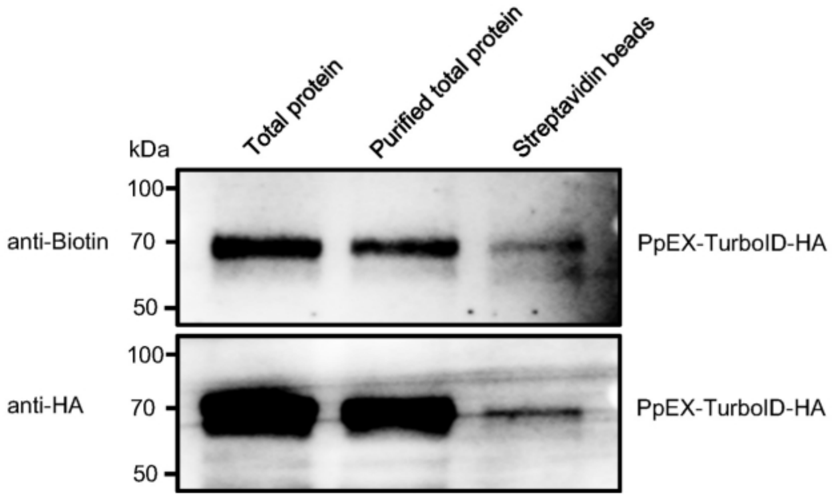

Figure S1. The expression and biotinylated modification detection of PpEX-TurboID-HA fusion protein. A single clear protein band corresponding to PpEX-TurboID-HA at 70 kDa, detectable using anti-biotin antibodies in the cell lysate, desalted lysate, and on Streptavidin beads. Additionally, anti-HA antibodies detected bands of the recombinant PpEX-TurboID-HA protein at the same location.

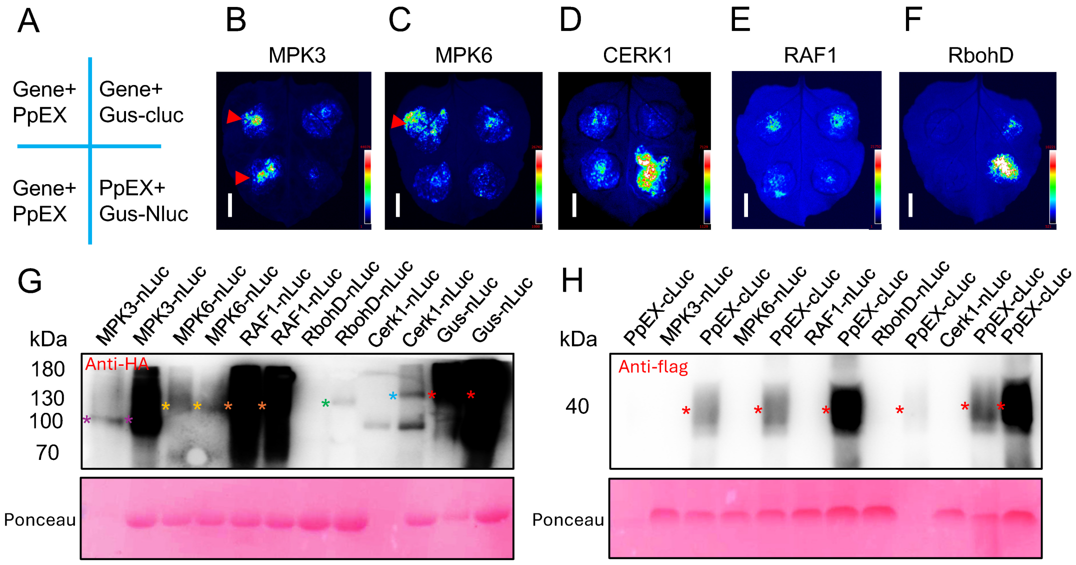

Figure S2. Split-Luciferase tests PpEX interactions with maize targets

(**A**) Schematic diagram of the luciferase detection assay. Gus-nLUC and Gus-cLUC serve as blank controls. Split-Luciferase verification results of PpEX with MPK3 (**B**), MPK6 (**C**), CERK1 (**D**), RAF1 (**E**), and RbohD (**F**). Western blot detection of expressed proteins with anti-HA (**G**) and anti-flag (**H**). The pentagram indicates the target protein.

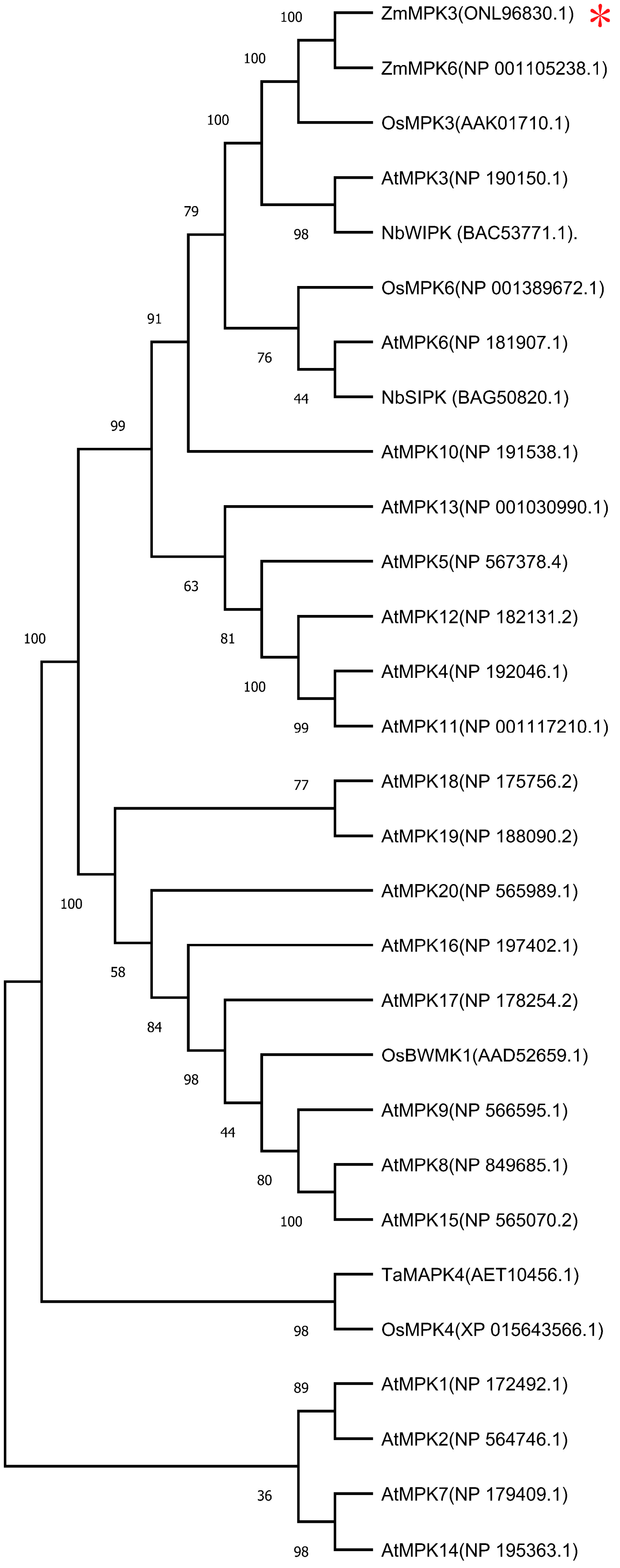
ZmMPK3(ONL96830.1)
ZmMPK6(NP 001105238.1)
OsMPK3(AAK01710.1)
AtMPK3(NP 190150.1)
NbWIPK (BAC53771.1).
OsMPK6(NP 001389672.1)
AtMPK6(NP 181907.1)
NbSIPK (BAG50820.1)
AtMPK10(NP 191538.1)
AtMPK13(NP 001030990.1)
AtMPK5(NP 567378.4)
AtMPK12(NP 182131.2)
AtMPK4(NP 192046.1)
AtMPK11(NP 001117210.1)
AtMPK18(NP 175756.2)
AtMPK19(NP 188090.2)
AtMPK20(NP 565989.1)
AtMPK16(NP 197402.1)
AtMPK17(NP 178254.2)
OsBWMK1(AAD52659.1)
AtMPK9(NP 566595.1)
AtMPK8(NP 849685.1)
AtMPK15(NP 565070.2)
TaMAPK4(AET10456.1)
OsMPK4(XP 015643566.1)
AtMPK1(NP 172492.1)
AtMPK2(NP 564746.1)
AtMPK7(NP 179409.1)
AtMPK14(NP 195363.1)

Figure S3. Phylogenetic analysis of the ZmMPK3 and other homologs by the maximum likelihood method.

Asterisk indicates this ZmMPK3 protein. The phylogenetic tree was constructed using MEGA 11 software. The sequences were referred to GenBank database.

Table S1. Primers used in this study

Primer name	Sequence (5’ to 3’)
SCMV-PpEX-HA-F	CATCAATCCGGACCTGGGCCCATGAGGACAGGTTTAATGGC
SCMV-PpEX-HA-R	ATGGAAAACATCTTCCCCGGGACCACATGACCGTAACTTGA
PUC19-PPEX-TURBOID- F	GAGAACACGGGGGACGAGCTCGGTACCATGAGGACAGGTTTAATGGCTAT
PUC19-PPEX-TURBOID- R	GTCTTTGCCACCTCCTGAACCGCCTCCACCACCACATGACCGTAACTTGA
PUC19-PPEX-GFP-F	TGGAGAGAACACGGGGGACGAGCTCATGAGGACAGGTTTAATGGCTAT
PUC19-PPEX-GFP-R	CTCCTTTACTCATCTCGAGGGTACCACCACATGACCGTAACTTGA
MPK3-1300-NLUC-F	ACGGGGGACGAGCTCGGTACCATGAGCGGAGGAGGCGTG
MPK3-1300-NLUC-R	AACATCGTATGGGTAGTCGACGTATCGGAAGTTGGGGTT
MPK6-1300-NLUC-F	ACGGGGGACGAGCTCGGTACCATGCAGCACGACCAGAAGAAG
MPK6-1300-NLUC-R	AACATCGTATGGGTAGTCGACGTATCGGAAGTTGGGGTT
CERK1-1300-NLUC-F	ACGGGGGACGAGCTCGGTACCATGGGGAACTGCTGGGGT
CERK1-1300-NLUC-R	AACATCGTATGGGTAGTCGACACGAGAATGGGCCAATGG
RAF1-1300-NLUC-F	ACGGGGGACGAGCTCGGTACCATGGGTATCGCTCTCCTC
RAF1-1300-NLUC-R	AACATCGTATGGGTAGTCGACCTCGCTGGAGTCTAGGCA
RBOHD-1300-NLUC-F	ACGGGGGACGAGCTCGGTACC ATGGCCCGCTTGGTCTCT
RBOHD-1300-NLUC-R	AACATCGTATGGGTAGTCGACTTCGCCGTTGATCTTCCT
PPEX-1300-CLUC-F	GAACACGGGGGACGAGCTCGGTACCATGAGGACAGGTTTAATGGCT
PPEX-1300-CLUC-R	CAGGAACATCGTATGGGTAGTCGACTTACTTATCGTCATCGTCCTTGTAA
